# Orf Virus Encoded Protein ORFV119 Induces Cell Apoptosis Through the Extrinsic and Intrinsic Pathways

**DOI:** 10.3389/fmicb.2018.01056

**Published:** 2018-05-29

**Authors:** Wei Li, Huiqin Chen, Hao Deng, Zhenzhan Kuang, Mingjian Long, Daxiang Chen, Xiaoqing Liao, Ming Li, Daniel L. Rock, Shuhong Luo, Wenbo Hao

**Affiliations:** ^1^Institute of Antibody Engineering, School of Laboratory Medicine and Biotechnology, Southern Medical University, Guangzhou, China; ^2^Guangdong Provincial Key Laboratory of Tropical Disease Research, School of Public Health, Southern Medical University, Guangzhou, China; ^3^Department of Pathobiology, College of Veterinary Medicine, University of Illinois at Champaign-Urbana, Urbana, IL, United States; ^4^Department of Laboratory Medicine, School of Stomatology and Medicine, Foshan University, Foshan, China

**Keywords:** parapoxvirus, orf virus, ORFV119, apoptosis, protein array

## Abstract

Apoptosis, a significant form of cell death, has a leading role in the host cell defense against virus infection. Viruses have evolved a series of strategies that block apoptosis during the early stage of viral infection to enhance viral replication, and induce apoptosis in the late stages to facilitate viral particle release from the cells. Here we show that orf virus (ORFV), the causative agent of orf, encodes an apoptosis-inducing protein ORFV119. ORFV119 targets the mitochondria in host cells, inhibits cell proliferation, and induces cell apoptosis. Protein array data indicated that ORFV119 could induce apoptosis via up-regulation of Smac, Bak, and Bax and down-regulation of anti-apoptotic proteins Bcl-2 and cIAP-2. Activation of caspase-9 and caspase-3, and consequent PARP cleavage, ultimately lead to apoptosis. ORFV119 could also directly activate caspase-8 and induce Bid, involved in the extrinsic pathway, to achieve cell death. Furthermore, sequence analysis and experiments with mutants of ORFV119 introduced revealed that ORFV119 contains a key N-terminal domain that is necessary and sufficient to direct the protein to the mitochondria. Together, we report, for the first time, the identification of the novel apoptosis-inducing protein ORFV119 encoded by a parapoxvirus. This provides an important reference for the study of pathogenesis, identification of immunomodulation mechanisms of ORFV, and may lead to new strategies for orf disease control.

## Introduction

The orf virus (ORFV), a member of the poxvirus family, primarily infects epithelial cells causing acute cutaneous pustular lesions or ecthyma contagiosum in sheep and goats (Haig and McInnes, 2002; Spyrou and Valiakos, 2015). ORFV occasionally infects camels (Azwai et al., 1995), Japanese serow (Inoshima et al., 2001, 2002), reindeer (Palatsi et al., 1993), and other animals (Fairley et al., 2008; Frandsen et al., 2011). In these natural hosts, the disease caused by ORFV is commonly known as contagious pustular dermatitis, scabby mouth, sore mouth, or orf (Hosamani et al., 2009). ORFV can also be transmitted to humans through broken skin, particularly in veterinarians, farmers, and animal caregivers in close contact with infected animals (Fleming et al., 2015; Thurman and Fitch, 2015). ORFV has a linear double-stranded DNA genome (134–139 kbp) with high GC content (approximately 63–64%), in comparison to other poxviruses (30–40%) (Wittek et al., 1979; Delhon et al., 2004), and encodes 132 putative gene products (Mercer et al., 2006). The core region of the central genome encodes proteins involved in the virus structure and assembly, and the terminal regions contain genes whose products are implicated in host range and virulence (Delhon et al., 2004). The viral particles consist of complete, ovoid-shaped virions with a crisscross-patterned tubule-like structure on the particle surface when viewed under atomic force microscopy (Li et al., 2012). Several highly variable ORFs (open reading frames) of the orf virus, ORFs 001, 103, 109–110, 116 and 132, were found using genomic sequence analysis. Interestingly, among the region of ORFs 118–120, the non-coding fragments are almost as long as the coding fragments (Li et al., 2015). According to this insight, we speculate that these areas might be part of the coding regions in early stages of orf virus evolution (Qiao et al., 2015). It is noteworthy, that ORFV119 was recently revealed able to inhibit NF-kappa B signaling through interaction with pRb (Nagendraprabhu et al., 2017).

Cell apoptosis, known as programmed cell death (PCD), plays a pivotal role in the body’s growth and development process (Meier et al., 2000). Apoptosis nominally occurs through two classic pathways, the exogenous pathway initiated by FasL, TRAIL or TNF (tumor necrosis factor), and the endogenous pathway triggered by stimuli such as cell-cycle dysregulation, survival factor withdrawal, drugs, DNA damage, or pathogen sensing (Duprez et al., 2009). Ultimately, both pathways result in the activation of caspases, subsequently releasing many apoptosis related proteins to cause cell death (Tait and Green, 2010). Apoptosis is not only closely related to the occurrence and development of many cancers or immune dysregulation, but also restricts virus replication in host cells (Veyer et al., 2014).

Through evolution, viruses have developed various strategies to confront host defenses. Early in the infection, viruses prevent apoptosis to facilitate replication within a receptive intracellular environment. However, in the late stages of infection, viruses induce apoptosis in order to spread the virus to adjacent cells or tissues (Collins, 1995; Razvi and Welsh, 1995). To counter host defenses, which could rapidly clear virally infected cells, many viruses express apoptosis-inhibiting factors. Proteins such as the myxoma virus encoded M11L protein (Everett et al., 2000; Su et al., 2008), vaccinia virus (VACV) encoded F1L protein (Wasilenko et al., 2003), deerpox virus encoded 022 protein (Banadyga et al., 2011), fowlpox virus encoded 039 protein (Banadyga et al., 2007), and ORFV encoded 125 protein (Westphal et al., 2007, 2009) have been shown to possess mitochondrial-targeting motifs and have anti-apoptotic functions. The investigation into ORFs 118–120 indicated that ORFV119 could be also confined to the mitochondria (Nagendraprabhu et al., 2017). Therefore, we hypothesized that ORFV119 may regulate cell apoptosis.

In the present study, we used multiple approaches to investigate whether the expression of ORFV119 can regulate apoptosis *in vitro* and seek related pathway and key targeted factors. Our findings indicated that ORFV119 could induce apoptosis through increasing pro-apoptotic proteins and suppressing expression of anti-apoptotic proteins.

## Materials and Methods

### Cells

Ovine fetal turbinate (OFTu) cells were cultured in Eagle minimal essential medium (MEM) (Gibco), while HeLa and 293T cells were grown in Dulbecco’s modified Eagle’s medium (DMEM) (Gibco, Grand Island, NY, United States). All media were supplemented with 10% fetal bovine serum (Hyclone Laboratories, Inc., South Logan, UT, United States), 1% penicillin–streptomycin (Sigma, St. Louis, MO, United States) and 2 mM L-glutamine (Gibco) and added to the cultures. All cells were incubated at 37°C in a humidified 5% CO_2_ atmosphere. Transient transfection was performed with X-tremeGENE9 Transfection Reagent (Roche, Basel, Switzerland) following the manufacturer’s instructions.

### Construction of Plasmids

To establish eukaryotic expression vectors, plasmids pEGFP-ORFV119 (p119GFP) and pCMV-ORFV119 (p119Flag) were constructed. ORFV119 full-length DNA was amplified using the IA-82 genome and cloned into the XhoI and Hind III sites of the vectors pEGFP-N1 and pCMV-Tag4. The primer sequences were synthesized as follows:

119GFP-Fw,   5′-CCGCTCGAGATGGACTCTCGTAGGC-3′; (XhoI)
119GFP-Rv,   5′-CCCAAGCTTATCGCTGTCGCTGTCG-3′; (Hind III)
119Flag-Fw,   5′-CCCAAGCTTATGGACTCTCGTAGGC-3′; (Hind III)
119Flag-Rv,   5′-CCGCTCGAGATCGCTGTCGCTGTCG-3′; (XhoI)

Restriction sites are underlined.

Plasmids p119GNmut1 and p119GNmut2 were constructed based on the pEGFP-N1 vector, while p119FNmut1 and p119FNmut2 were constructed based on the pCMV-Tag4 vector. The proteins expressed by these recombinant vectors were respectively named as 119Nmut1 (carrying either tag), 119GNmut1 (GFP tagged), 119FNmut1 (Flag tagged), 119Nmut2 (carrying either tag), 119GNmut2 (GFP tagged) and 119FNmut2 (Flag tagged).

The primer sequences were synthesized as follows:

119GNmut1-Fw,   5′-CCGCTCGAGATGACACAGCGCC-3′; (XhoI)
119GNmut1-Rv/119GNmut2-Rv,   5′-CCCAAGCTTATCGCTGTCGCTGTCG-3′; (Hind III)
119FNmut1-Fw,   5′-CCCAAGCTTATGACACAGCGCC-3′; (Hind III)
119FNmut1/119FNmut2-Rv,   5′-CCGCTCGAGATCGCTGTCGCTGTCG-3′; (XhoI)
119GNmut2-Fw,   5′-CCGCTCGAGATGGGCGGCGACG-3′; (XhoI)
119FNmut2-Fw,   5′-CCAAGCTTATGGGCGGCGACG-3′; (Hind III)

Restriction sites are underlined.

### Fluorescence Microscopy

HeLa/OFTU cells were co-transfected with either p119GFP or pEGFP-N1 + pMitoDsRed (used as a mitochondrial marker) or pDSRed-Mito (used as an endoplasmic reticulum marker). After 24 h, discarding the medium, cells were washed twice with PBS and fixed with 4% paraformaldehyde solution for 15 min at room temperature, PBS washed twice for 5 min, then the cells were stained with 4,6,-diamidino-2-phenylindole (DAPI, 0.1 ug/ml) at room temperature, away from light for 20 min. The cells were observed under the laser scanning confocal microscope (LSM700; Zeiss, Germany).

### Cell Proliferation Assay

The 293T cells were cultured in 96-well plates, until approximately 40% confluent. The next day, the cells were transfected with different plasmids. After 24, 48, 72 h, CCK8 solution (Dojindo, Japan) was added into the culture medium at a 1:10 dilution. At each time point, the absorbance at 450 nm was determined using an iMark microplate reader (Bio-Rad, Hercules, CA, United States).

### Caspase Activity and Inhibitor Assays

Caspase-3, caspase-8, and caspase-9 activities in 293T cells were measured using caspase colorimetric assay kits (Keygen, Nanjing, Jiangsu, China), according to manufacturer’s protocol. Transfected cells were collected by centrifugation. The cells were washed twice with PBS and lysed using ice-cold lysis buffer for 40–60 min. Lysates were centrifuged at 1000 rpm for 1 min, after which 2× reaction buffer and caspase substrate were added into the supernatant and incubated in the dark for 4 h at 37°C. The luminescent signal was measured at 400 nm using an iMark microplate reader (Bio-Rad). For the inhibitor assay, cells were pre-treated with 50 μM caspase-8 inhibitor (Z-IETD-FMK) or caspase-9 inhibitor (Z-LEHD-FMK) for 1 h. The cells were transfected with p119GFP for 24 h, and the caspase-3, caspase-8, and caspase-9 assays were performed.

### Flow Cytometry

The effect of apoptosis-induction was confirmed using the Apoptosis Detection Kit (Keygen, Nanjing, Jiangsu, China), according to the manufacturer’s protocol. Briefly, cells for transfection were detached with enzyme-free EDTA, washed twice with PBS and suspended at a concentration of 5×10^5^ cells in 300 μL binding buffer. Annexin V-APC and 7-AAD were added to the cells and allowed to incubate for 15 min at room temperature in the dark. Apoptotic cells were identified using an Accuri C6 flow cytometer (BD Biosciences, San Jose, CA, United States) on the FL-4 and FL-3 channels. Data were analyzed using the Flow Express software (De Novo Software, Los Angeles, CA, United States).

### TUNEL Assay

As a classic method, the TUNEL (Terminal-deoxynucleoitidyl Transferase Mediated Nick End Labeling) assay was used to detect apoptosis. The Hela cells were seeded in six-well plates at a density of 5 × 10^5^ cells per well and incubated at 37°C in 5% CO_2_. The following day, the cells were transfected with pEGFP-N1 or p119GFP for 24 h. Apoptotic cells induced by ORFV119 were measured qualitatively by labeling (TUNEL) assay (KeyGen, Nanjing, China), according to the manufacturer’s instructions. The TUNEL assay was repeated at least three times.

### Protein Microarray

The 293T cells transfected with pEGFP-N1, p119GFP or p119GNmut2 were evaluated for apoptosis-related proteins using Human Apoptosis Antibody Microarray slides (RayBiotech, Norcross, GA, United States), according to the manufacturer’s instructions. Briefly, the total proteins of cells were extracted using cell lysis buffer (RayBiotech, Norcross, GA, United States) 24 h after transfection. Proteins from each sample were incubated with the human apoptosis array overnight. After being washed and incubated with a biotin-conjugated anti-cytokine mix, the slides were scanned with an ImageQuant LAS4000 Scanner (GE Healthcare, Chicago, IL, United States). Analysis of the signal values was performed using the RayBiotech analysis tool and SPSS 20.0 software (IBM, Corp., Armonk, NY, United States).

### Protein Extracts and Immunoblotting

The total proteins of 293T cells were extracted with lysis buffer [20 mM Tris-HCl, 137 mM NaCl, 10% glycerol, 1% Triton X-100, 2 mM EDTA, supplemented with 0.1% PMSF (Beyotime, China)]. The lysates were shaken on ice for 15 min, and the protein concentration was ascertained by BCA assay. The cell lysates were mixed with 2× SDS sample loading buffer and subjected to 10% or 12% (dependent on predicted protein molecular weight) SDS polyacrylamide gel electrophoresis analysis. The proteins were transferred to a PVDF membrane. After blocking with 5% skim milk in TBS-T (0.12 M Tris-base, 1.5 M NaCl, 0.1% Tween-20), the membrane was incubated with primary antibodies, targeting Cleaved-caspase-3, Cleaved-caspase-9, Cleaved-caspas-8, Cleaved-PARP, Bax, Bak, Bid, Smac/Diablo, cIAP-2, Bcl-2, and Rb (all purchased from Cell Signaling Technologies, Danvers, MA, United States), at 4°C overnight. The blots were washed three times in TBST (20 mM pH 7.4 Tris-HCl, 150 mM NaCl, 0.05% Tween-20) and incubated with secondary goat anti-rabbit/mouse horseradish peroxidase-conjugated IgG. The membranes were washed thrice with TBST and the protein was visualized using an ECL chemiluminescent substrate according to the manufacturer’s instructions (Pierce-Thermo Fisher Scientific, Waltham, MA, United States).

### ELISA Assay

Briefly, 293T cells were transfected with pEGFP-N1 or p119GFP for 24 and 48 h. The culture media was removed and the cells were rinsed with ice-cold PBS. Cells were scraped off the plates and transferred to appropriate tubes. Cell suspensions were diluted with 1x PBS and stored overnight at -20°C. The cell membranes were ruptured after two freeze-thaw cycles and the cell lysates were centrifuged for 5 min at 5000 ×*g* at 4°C. The supernatant was then collected. Cyt-C and TNF-α were detected using Human Cytochrome C ELISA kit (CUAABIO, Wuhan, China) and Human Tumor Necrosis Factor α ELISA kits (CUAABIO, Wuhan, China), according to the manufacturer’s instructions.

### Statistical Analyses

Experimental data were presented as the means ± standard deviation from at least three independent experiments. The *t*-test or one-way ANOVA accompanied by *post hoc* Dunnett’s test were performed to compare differences between the groups by using SPSS 20.0 software (IBM, Corp., Armonk, NY, United States). *P* < 0.05 was considered statistically significant.

## Results

### ORFV119 Localizes in the Mitochondria Dependent on N-Terminal Domain

**FIGURE 1:**
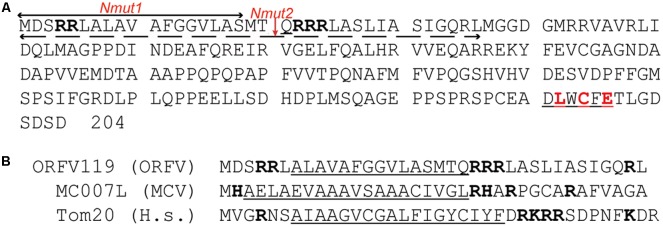
Analysis of ORFV119 amino acid sequence. **(A)** ORFV119 amino acid sequence. The N-terminal region contains the transmembrane segments on both sides of the basic amino acid (black body); C-terminal region contains the pRb binding motif (LxCxE: red). The deleted fragment in recombinant protein 119Nmut1 is marked with a line above and named as Nmut1. The mutant fragment in 119Nmut2 is underlined with a dotted line and named as Nmut2. **(B)** Homology comparison of N-terminal sequences of ORFV119, MCVMC007L, and human Tom20. The mitochondrial signal targeting peptide is underlined.

The ORFV119 gene of the IA82 strain contains 715 nucleotides and encodes the 204 amino acid ORFV119 protein, described in previous study (Nagendraprabhu et al., 2017) (**Figure 1A**). This study also predicted a N-terminus sequence fragment (RRLALAVAFGGVLASMTQRRR) related to mitochondrial localization which is found in most ORFV strains and PCPV (Nagendraprabhu et al., 2017). Through extra bioinformatic analysis, this transmembrane hydrophobic sequence at the N-terminal segment of ORFV119 was determined to be highly homologous with the sequence of the human Tom20 protein and the molluscum contagiosum virus (MCV) MC007L protein (**Figure 1B**). Thus, we speculated that ORFV119 could localize in mitochondria.

**FIGURE 2:**
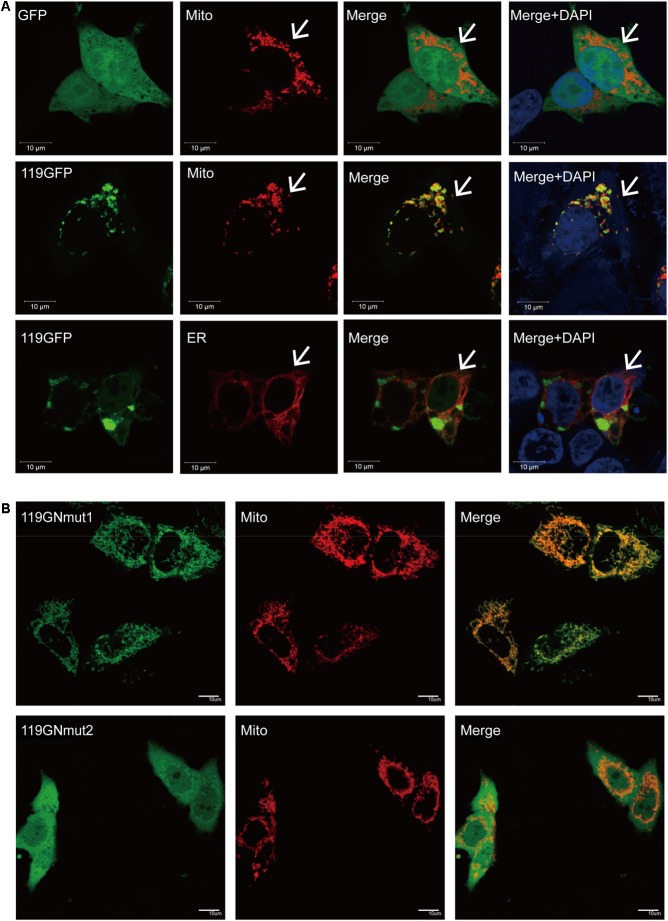
Cellular localization of ORFV119. **(A)** ORFV119 localizes in the mitochondria. OFTu cells were transfected with pEGFP-N1/p119GFP + pMitoDsRed or pERDsRed, fixed, stained and observed under laser confocal fluorescence microscopy after 24 h. GFP and 119GFP are shown in green; Mitochondria (Mito) are red; Endoplasmic reticula (ER) are shown in red. Nuclei were DAPI stained and are shown in blue. ORFV119 co-localized in the mitochondria and appeared yellow when merged with mitochondria marker; arrows denote the mitochondria. **(B)** The intracellular location of 119Nmut1/119Nmut2. OFTu cells were co-transfected with p119GNmut1/p119GNmut2 and pMitoDsRed. Colocalization was visualized by confocal microscopy.

The cellular localization of ORFV119 was investigated by immunofluorescence analysis 24 h after co-transfection with pEGFP-N1/p119GFP + pMitoDsRed or pERDsRed in OFTu cells. The results confirmed that the ORFV119 protein localizes in the mitochondria (**Figure 2A**). To study further the functional domain of ORFV119 related to intracellular localization, plasmids p119GNmut1 and p119GNmut2 were constructed to express 119Nmut1 or 119Nmut2, two kinds of N-terminal mutants of ORFV119 which varied by different coding start-positions (**Figure 1A**). They were shortened by 18 and 36 amino acids, compared with intact ORFV119. HeLa cells were transfected with multiple plasmids and the orientation changes were observed using a confocal microscope. ORFV119 localized to the mitochondria, as observed in the OFTu cells, proving that the cellular localization is identical in both human or sheep epithelial cells (**Supplementary Figure S1**). After transfection with p119GNmut1, localization did not change. However, when HeLa cells were transfected with p119GNmut2, the fluorescence was uniformly distributed throughout the cell rather than being restricted to the mitochondria (**Figure 2B**). Collectively, these data revealed that ORFV119 localizes in the mitochondria, and its intracellular localization will change when its first 36 amino acids in N-terminal were deleted, which indicates this N-terminal domain is vital for intracellular signaling.

### ORFV119 Induces Cell Apoptosis

**FIGURE 3:**
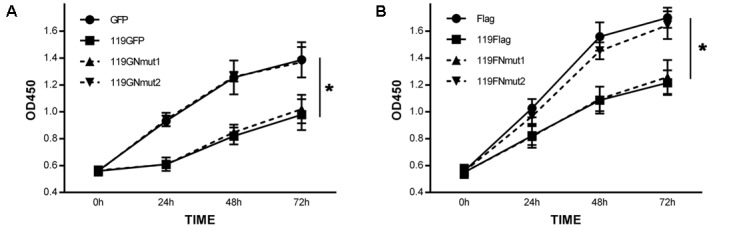
Inhibition of cell proliferation by ORFV119. **(A)** 293T cells were transfected with pEGFP-N1, p119GFP, p119GNmut1or p119GNmut2. **(B)** 293T cells were transfected with pCMV-tag4, p119Flag, p119FNmut1 or p119FNmut2. The OD450 values were determined using Cell Counting Kit 8 at 0 h, 24 h, 48 h, and 72 h. Cell growth curves were plotted. Data are presented as the average of three independent experiments performed in duplicate. ^∗^*P* < 0.05.

To evaluate the effect of ORFV119 on cell proliferation, cell growth curves were determined. The absorbance of cells was lower in ORFV119-overexpressing cells compared with cells transfected with vectors only (**Figure 3**, *P* < 0.05). These data indicated that ORFV119 could inhibit cell proliferation. Further, when plasmids expressing 119Nmut1 and 119Nmut2 (with either GFP or Flag tagged) were used in cell proliferation assay, we found out only the latter mutant can influence the inhibition of cell proliferation comparing with ORFV119 (**Figure 3**, *P* < 0.05).

**FIGURE 4:**
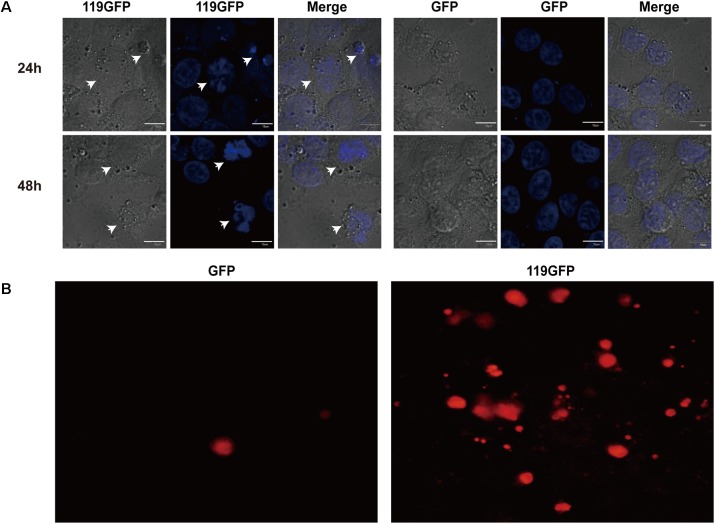
ORFV119 induced apoptosis as evidenced by morphologic changes and TUNEL assay. Cells were transected with pEGFP-N1 or p119GFP. **(A)** 24 h after transfection, the morphological changes of cells were observed by fluorescent microscopy (400×). For nuclear morphology analysis, cells were fixed, stained with DAPI for 30 min, and analyzed. Arrowheads showing apoptotic cells. **(B)** 24 h after transfection, ORFV119-induced apoptotic cells were determined qualitatively by labeling (TUNEL) assay.

**FIGURE 5:**
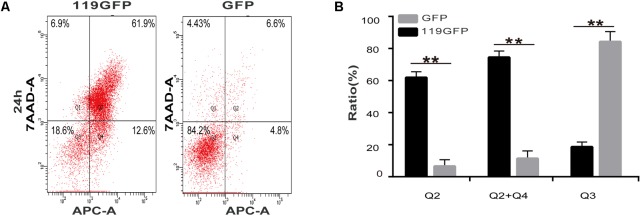
Flow cytometry analysis of ORFV119-induced apoptosis. 293T cells were transfected with pEGFP-N1 or p119GFP for 24 h. Cells were collected, washed and dyed with Annexin V-APC and 7-AAD. **(A)** Apoptotic cells were detected using a flow cytometer (Q3: the normal cells; Q2: late apoptotic cells; Q2 + Q4: overall apoptotic cells). **(B)** Data are presented as mean ± standard deviation. ^∗∗^*P* < 0.01 as compared with control.

Due to its intracellular distribution, we speculated that ORFV119 can affect apoptosis. To investigate this hypothesis, multiple methods were used to detect apoptosis reliably. As shown in **Figure 4A**, characteristic nuclear pyknosis and fission were observed under fluorescent microscopy in HeLa cells transfected with p119GFP. The TUNEL assay showed cell apoptosis significantly increased when transfected with p119GFP, compared to the GFP groups (**Figure 4B**). Flow cytometry analysis showed that expression of ORFV119 in 293T cells led to a remarkable increase in the rate of late apoptotic cells (double stained, Q_2_) and overall apoptotic cells (Q_2_+Q_4_) (**Figure 5**, *P* < 0.01).

### ORFV119 Induces Activation of Various Caspases-3, -9, and -8

**FIGURE 6:**
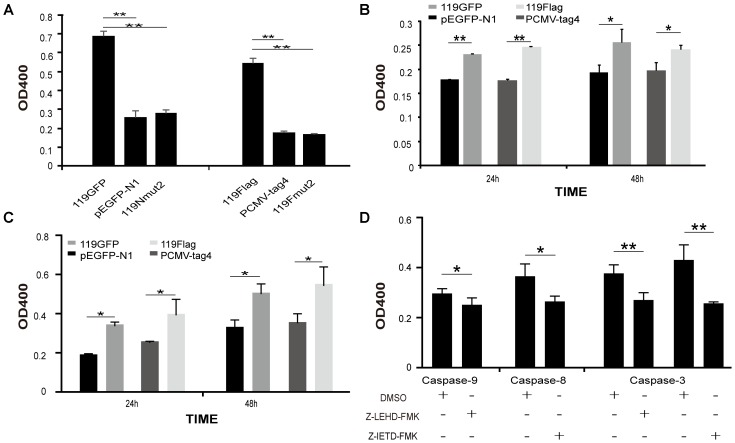
Caspase-3, caspase-8, and caspase-9 were activated by ORFV119. **(A–C)** 293T cells were transected with vectors able to express ORFV119 or 119Nmut2, as well as pEGFP-N1 or pCMV-tag4 as mock controls. The cells were washed, lysed and caspase substrate was added. Caspase-3, caspase-8, and caspase-9 activities were measured using a micro-plate reader (Bio-Rad). **(D)** Cells were pre-treated with 50 μM caspase-8 inhibitor (Z-IETD-FMK) or caspase-9 inhibitor (Z-LEHD-FMK) for 1 h. The cells were transfected with p119GFP or pEGFP-N1 for 24 h, and the caspase-3, caspase-8, and caspase-9 activites were measured as above. The data shown in the panels are averages of three independent experiments with standard deviations indicated. ^∗^*P* < 0.05, ^∗∗^*P* < 0.01.

Because they play critical roles in various apoptotic pathways, caspase-3, caspase-8, and caspase-9 were monitored to identify pathways possibly regulated by ORFV119. 293T cells transiently expressing ORFV119 showed a sharp increase in caspase-3 activity at 24 h after transfection, compared to other groups (*P* < 0.01). There were no significant differences between the pEGFP-N1 group and p119GNmut2 group, or between the pCMV-tag4 group and the p119FNmut2 group (**Figure 6A** and **Supplementary Figure S2**). The results indicated that ORFV119 could induce cell death, while 119Nmut2 could not, a difference likely attributed to intracellular distributions.

Similarly, the activities of caspase-8 and caspase-9 in 293T cells were measured after transient expression of ORFV119, and significant deviations compared to the control groups were observed at 24 h (*P* < 0.01) and 48 h (*P* < 0.05) (**Figures 6B,C**). In addition, pre-treatment with either caspase-8 inhibitor (Z-IED-FMK) or caspase-9 inhibitor (Z-LEHD-FMK) followed by the ORFV119 transient expression resulted in lower caspase-3 activation than in cells without pre-treatment (**Figure 6D**). These results suggested that ORFV119 can initiate apoptosis through intrinsic and extrinsic pathways.

### ORFV119 Regulates the Expression of Multiple Proteins in the Process of Apoptosis by Protein Chip Technology

**FIGURE 7:**
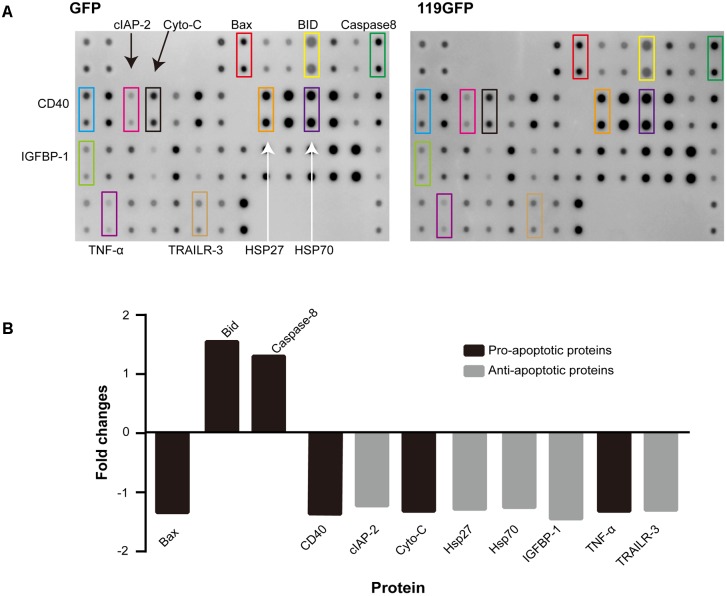
Human apoptotic protein array analysis of multiple proteins regulated by ORFV119. 293T cells were transfected with pEGFP-N1 or p119GFP, and apoptosis-related proteins were detected and analyzed using Human Apoptosis Antibody Microarray slides. **(A)** Characteristic proteins detected in antibody array. **(B)** Fold changes (ratio of medians among groups) of apoptotic signaling molecules, in comparison to controls, with a cutoff limit of 1.25 fold.

To explore the mechanisms and potential targets of apoptotic regulation by ORFV119, we performed an antibody array which can simultaneously detect expression changes in 43 intracellular and secreted proteins associated with apoptosis. Because there were almost no differences in the apoptosis array results between the ORFV119-treated group and the 119Nmut1-treated group, only the ORFV119-treated group and control group were further analyzed and verified. Of the detected proteins, pro-apoptotic proteins Bid and Caspase-8 were up-regulated. Meanwhile, five anti-apoptotic proteins, including cIAP-2, HSP27, HAP70, IGFBP-1 and TRAILR-3, were reduced when ORFV119 was overexpressed in 293T cells (**Figure 7**). Taken together, these results showed that both intrinsic and extrinsic apoptotic pathways were involved in the process of ORFV119-induced apoptosis.

### ORFV119 Up-Regulates Multiple Pro-apoptotic Proteins and Down-Regulates Expression of Multiple Apoptotic Inhibiters

**FIGURE 8:**
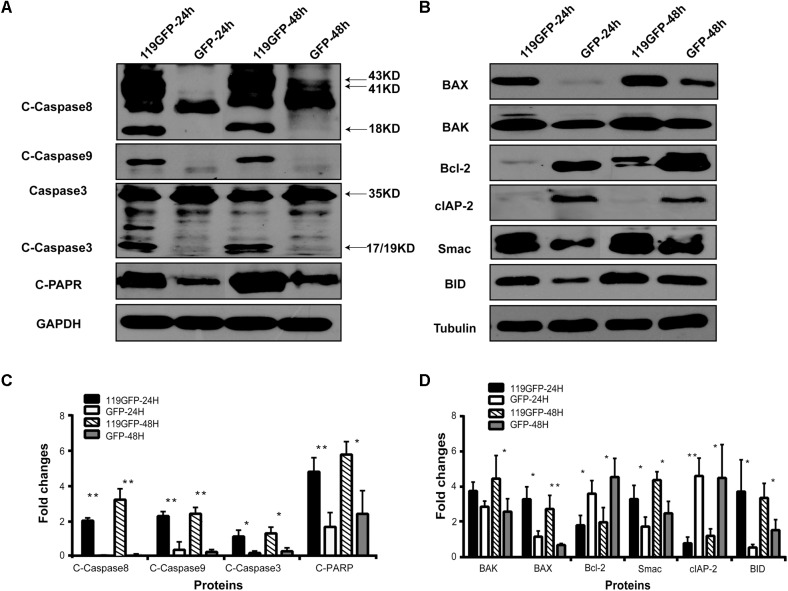
ORFV119 upregulated apoptotic promoters and downregulated apoptosis inhibitors. 293T cells were transfected with p119GFP or pEGFP-N1 for 24 h and 48 h. The protein expression levels of cleaved-caspase-3, cleaved-caspase-8, cleaved-caspase-9, cleaved-PARP, Bax, Bak, Bcl-2, Smac, cIAP-2, and BID were assessed by western blot using relevant primary antibodies. GAPDH/Tubulin served as the loading controls. Data are shown as mean ± SD of three independent experiments. ^∗^*P* < 0.05, ^∗∗^*P* < 0.01.

**FIGURE 9:**
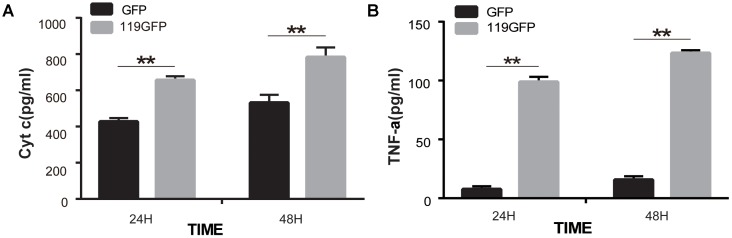
ORFV119 promoted the release of Cyto C and TNF-α. 293T cells were transfected with p119GFP or pEGFP-N1 for 24 h and 48 h. Cell lysates were collected and Cyto C and TNF-α were detected using a Human Cytochrome C/Tumor Necrosis Factor ELISA kit. The experiments were repeated three times with similar results. ^∗∗^*P* < 0.01.

Immunoblotting and ELISA assays were used to identify the regulation of molecules by ORFV119. High expression of ORFV119 was shown to trigger mitochondrial-mediated apoptotic pathways in eukaryotes by activating caspase-9, caspase-3 and PARP, and by up-regulation of the expression of pro-apoptotic proteins Bax, Bak, and Smac. Concurrently, anti-apoptotic proteins Bcl-2 and cIAP-2 were down-regulated (**Figures 8A,B**). ORFV119 was also able to active extrinsic apoptotic pathway by up-regulating the activation of caspase-8 and Bid (**Figure 8**). The results of the ELISA assay indicated Cyto C and TNF-α were significantly released in the ORFV119 groups, compared with the control, at 24 and 48 h (*P* < 0.05) (**Figure 9**).

## Discussion

The relationship between virus and the host is complicated. Viruses rely on healthy host cells for their survival, proliferation and propagation, but the host must remove infected cells to keep itself in prime condition (Roulston et al., 1999; Taylor and Barry, 2006; Neumann et al., 2015). Therefore, it is vital for viruses to suppress host cell apoptosis by stimulating anti-apoptotic pathways at the early stages of infection, thereby evading premature cell death and producing progeny virus. On the other hand, viruses cause cell death to promote the spread of viral particles into other cells or adjacent tissues at the late stages of infection by breaking down infected cells (Hardwick, 1997). Many viral proteins modulate biphasic apoptosis during viral infection (Yatim and Albert, 2011; Herold et al., 2012). Type 1 PRRSV (Costers et al., 2008) and type 2 PRRSV (Yuan et al., 2016) have been demonstrated to possess a dichotomous apoptosis-regulating role through inhibiting apoptosis early in infection, but inducing apoptosis late in infection. In the present study, the results revealed that ORFV119 localizes to the mitochondria and the N-terminal domain had a vital role in the localization of ORFV119. Many mitochondrion-targeted proteins have been correlated with apoptosis, such as M11L (Everett et al., 2000; Su et al., 2008), F1L (Wasilenko et al., 2003), and ORFV125 (Westphal et al., 2007, 2009). In a recently published study, the data suggested the ORFV119 may perform functions in addition to the early virion-associated NF-kappaB inhibitory function described, and a mitochondrial localization sequence was predicted (Nagendraprabhu et al., 2017). Therefore, in the present article, we set out to test the hypothesis that ORFV119 can modulate apoptosis and to explore further the potential proximate mechanism.

Apoptosis is characterized by cell morphological changes, such as cytoplasmic shrinkage, plasma membrane blebbing, chromatin condensation, nuclear fragmentation, and subsequently formed apoptotic bodies in the membrane-enclosed vesicles, which are then devoured by neighboring cells or phagocytes (Wong, 2011). Through immunofluorescence, nucleus shrinkage, and fragmentation has been observed. DNA fragments of apoptotic cells integrated with dUTP and appeared red, and significant cell apoptosis was observed by TUNEL assay (**Figure 4**). Flow cytometry analysis and caspase activation assays further corroborated evidence for apoptosis-regulation by ORFV119.

To trace the domain related to attribution and subsequent function, we constructed two kinds of mutants of ORFV119, namely 119Nmut1 and 119Nmut2. Cells transfected with p119GNmut2/ p119FNmut2 (expressing 119Nmut2) could not active caspase-3, while p119GFP/p119Flag transfected cells were able to do so (**Figure 6A**). When the cells were transfected with p119GFP or p119GNmut1 (expressing 119Nmut1), the fluorescence was confined in the mitochondria. However, the fluorescence was distributed throughout the cell after transfecting p119GNmut2 (**Figure 2**). Besides, when transfected with plasmids expressing ORFV119 or 119Nmut1, cell proliferations were inhibited comparing with control, but when transfected with plasmids expressing 119Nmut2, the cell proliferations were not affected (**Figure 3**). The difference between 119Nmut1 and 119Nmut2 was the length of mutant fragments. The mutant fragment in 119Nmut2 is 18 amino acids longer than which in 119Nmut1, which only have the first 18 amino acids in N-terminal been deleted (**Figure 1A**). Interestingly, the mitochondrial signal targeting peptide (**RR**LALAVAFGGVLASMTQ**RRR**), proposed in previous prediction (Nagendraprabhu et al., 2017) and extra bioinformatics analysis (**Figure 1B**), spans both the first 18 and the second 18 amino acids. But only when the whole peptide was deleted, the intracellular localization would change. This indicates that incomplete peptide (MTQ**RRR**) would also function for targeting in this case, perhaps with the help of other fragments in the second 18 amino acids.

There are three principal apoptotic pathways. The intrinsic apoptotic pathway is triggered by intracellular stresses and is strictly regulated by Bcl-2 family proteins. The extrinsic pathway relates to the binding of cell surface death receptors (Fas/APO-3L/TRAIL/TNF) with their respective ligands (Tzifi et al., 2012). Finally, there is also the endoplasmic reticulum (ER) stress pathway (Majors et al., 2007). All of these pathways will eventually promote the activation of the caspase cascade, which then triggers a sequential series of biochemical events that lead to cell changes and death (Elmore, 2007). The mechanism by which apoptosis is regulated is determined by both virus and cell type. For instance, the VSV-GFP virus activates apoptosis via both the intrinsic and extrinsic pathways in most PDAC cell lines, but only the intrinsic pathway in Capan-2 and AsPC-1 cell lines (Felt et al., 2015). Earlier reports revealed pro- and anti-apoptotic proteins/drugs involved in the extrinsic, intrinsic and/or the ER stress pathway, such as Nsp4 and Nsp10 encoded by PRRSV (Yuan et al., 2016), Curcumin (Zeng et al., 2016), Goniothalamin (Li et al., 2016). Several viruses, such as herpes simplex virus, Varicella zoster virus, rabies virus, human immunodeficiency virus, and reovirus, have been shown to induce apoptosis in susceptible cells (Kennedy, 2015). It is worth noting that an ORFV-encoded ankyrin-repeat (AR), ORFV126 has been identified to target the mitochondria of ORFV-infected cells and cells transiently expressing ANK-1, although it is not obviously related to apoptosis regulation (Lacek et al., 2014). Thus, in further studies, we will investigate the interaction of ORFV119 with other ORFV-encoded proteins which are involved in the apoptosis regulation pathway or localize to mitochondria.

**FIGURE 10:**
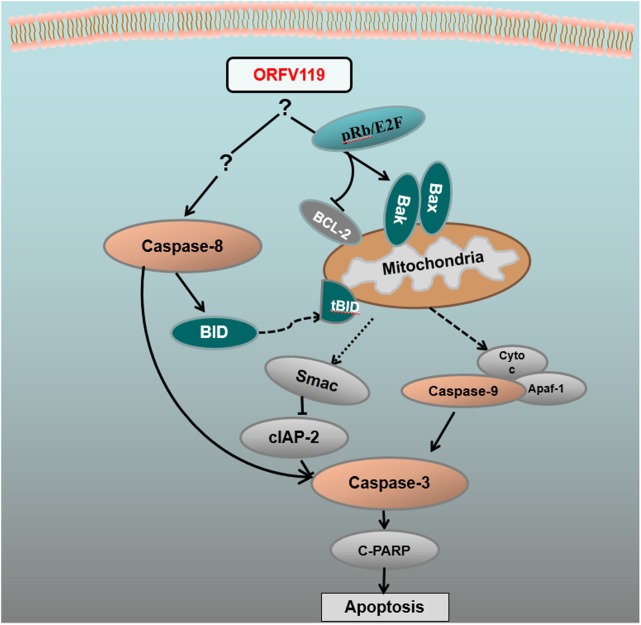
Pro-apoptotic network map of ORFV119. Caspase-3, caspase-8, and caspase-9 were activated in the current study. Expression of BID, Bax, Bak, Smac, and cleave-PARP were upregulated, while expression of BCL-2 and cIAP-2 were inhibited. Apoptosis was ultimately induced.

In the present study, we used the apoptosis array assay to screen correlated cytokines in the apoptotic pathways. The discrepancies were not visually significant, so signal analysis using specific instruments and subsequent verification including western blot and other experiments are very necessary. The apoptosis array data and western blot analysis revealed that ORFV119 manipulated apoptosis primarily through the intrinsic pathway, although some participation of the extrinsic pathway was involved (**Figure 10**). ORFV119 increased the expression of Bax and Bak and the degradation of anti-apoptotic proteins Bcl-2 and cIAP-2 (BIRC3). Bax and Bak perturbed mitochondrial membrane potential and the subsequent release of cytochrome C and Smac from the mitochondrial intermembrane space into the cytoplasm, as well as cleaved procaspase-9. Activated caspase-9, along with Apaf-1 and Cyto-c, formed a complex known as apoptosis bodies that activate caspase-3 and PARP and cause cells to die (**Figures 8**, **10**). Smac/Diablo, the second mitochondria-derived activator of caspase, is capable of directly binding cIAP-1/2 to induce their auto-ubiquitination and degradation to promote apoptosis (Du et al., 2000; Yang and Du, 2004). ORFV119 also directly activated caspase-8 and Bid, the pro-apoptotic protein of the BH3-only proteins. Bid usually exists in an inactive form in the cytosolic fraction of living cells and is cleaved or activated by caspase-8 at the onset of apoptosis. The tBid, a form of cleaved Bid, connects the intrinsic pathway and the extrinsic pathway by activating Bax, Bak, and the release of cytochrome C (Li et al., 1998; Kaufmann et al., 2012). Cells become apoptotic when the effect of pro-apoptotic proteins surpasses that of anti-apoptotic proteins at the mitochondrial membrane in the intrinsic pathway (Tomita, 2016).

In brief, we provide new insight into the pathway by which viruses modulate apoptosis-associated proteins. However, further research is necessary to fully characterize this process, such as identifying the functional domain of ORFV119. Because some studies have reported that the deletion of the ORF119 gene had no significant effect on viral replication and virulence (Qiao et al., 2015), future studies should include more ORFV strains, since virulence and genomes vary greatly among ORFV strains, viral recombination and subsequent *in vivo* experiments (Delhon et al., 2004; Chi et al., 2015; Li et al., 2015). In addition, the relevance and interaction between regulation of NF-κB and regulation of apoptosis need to be explored further (Nagendraprabhu et al., 2017).

In summary, this report indicates that ORFV119 represents a novel protein that poxviruses have evolved to induce cell death via down-regulation of the anti-apoptotic proteins cIAP-2 and Bcl-2, and up-regulation of the pro-apoptotic proteins Bax, Bak, and Smac. These results could provide implications for elucidating the mechanisms of invasion, survival, reproduction, and spread of ORFV, and contribute to the prevention and treatment of ORFV and analogous virus infections.

## Author Contributions

WL, SL, and WH participated in design of the study. WL, HC, DC, HD, ZK, and MJL contributed reagents, materials, and analysis tools. WL, HC, HD, XL, and MJL performed the experiments. WL, HC, ML, DR, and SL analyzed the experimental data. WL, DR, SL, and WH wrote and edited the manuscript. All authors have reviewed the manuscript.

## Conflict of Interest Statement

The authors declare that the research was conducted in the absence of any commercial or financial relationships that could be construed as a potential conflict of interest.

## Supplementary Material

FIGURE S1 ORFV119 protein is localized in the mitochondria. HeLa cells were transfected with pEGFP-N1/p119GFP + pMitoDsRed for 24 h, fixed, stained and observed under laser confocal fluorescence microscopy.

FIGURE S2 ORFV119 protein was successfully expressed after transfection.
