# Supplementary figures and images for: Orf Virus Encoded Protein ORFV119 Induces Cell Apoptosis Through the Extrinsic and Intrinsic Pathways
Source: Front Microbiol. 2018 May 29;9:1056. doi: 10.3389/fmicb.2018.01056 (PMC5986898; doi:10.3389/fmicb.2018.01056)

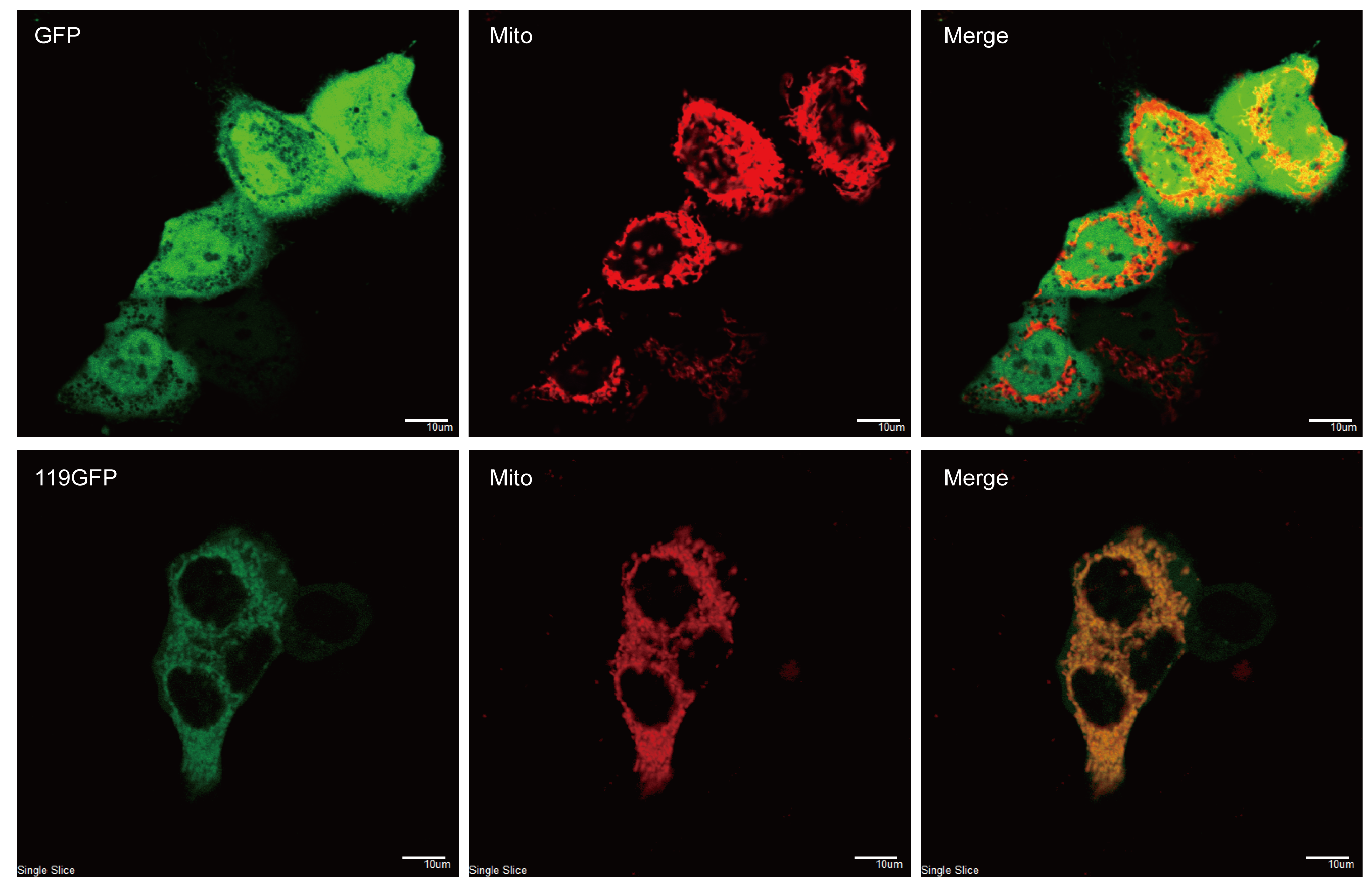

Supplement: FIGURE S1 — ORFV119 protein is localized in the mitochondria. HeLa cells were transfected with pEGFP-N1/p119GFP + pMitoDsRed for 24 h, fixed, stained and observed under laser confocal fluorescence microscopy. [file Image_1.TIF]

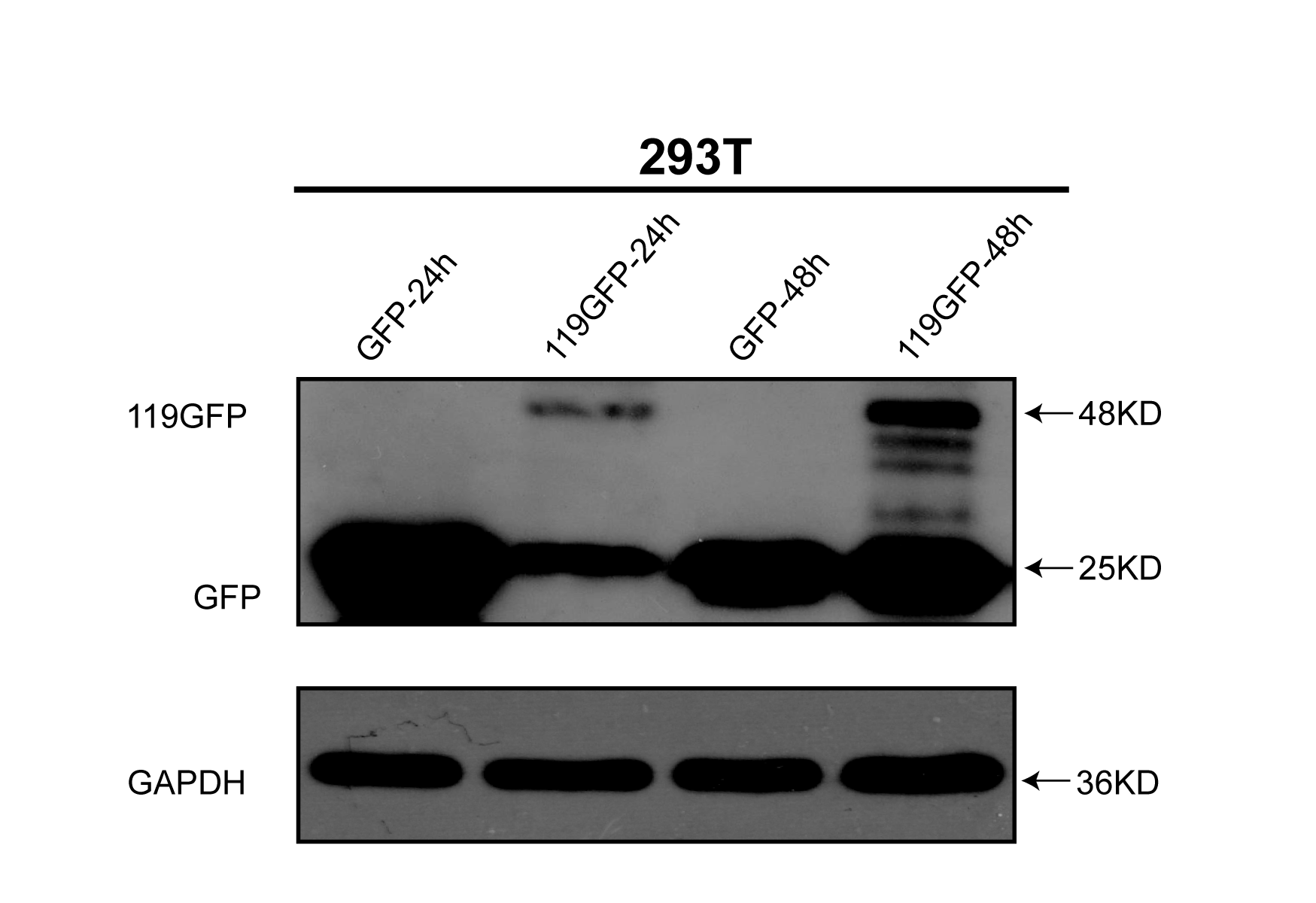

Supplement: FIGURE S2 — ORFV119 protein was successfully expressed after transfection. [file Image_2.TIF]
